# Post-stroke depression: exploring gut microbiota-mediated barrier dysfunction through immune regulation

**DOI:** 10.3389/fimmu.2025.1547365

**Published:** 2025-03-03

**Authors:** Jia Jiang, Haihua Xie, Sihui Cao, Xuan Xu, Jingying Zhou, Qianyan Liu, Changsong Ding, Mi Liu

**Affiliations:** ^1^ The Second Affiliated Hospital, Hunan University of Chinese Medicine, Changsha, China; ^2^ School of Acupuncture & Tuina and Rehabilitation, Hunan University of Chinese Medicine, Changsha, China; ^3^ School of Information Science and Engineering, Hunan University of Chinese Medicine, Changsha, China

**Keywords:** post-stroke depression, ischemic stroke, gut microbiota, immune regulation, barrier integrity and function, microbiota-gut-brain axis, inflammatory response, microbiota-immune-barrier axis

## Abstract

Post-stroke depression (PSD) is one of the most common and devastating neuropsychiatric complications in stroke patients, affecting more than one-third of survivors of ischemic stroke (IS). Despite its high incidence, PSD is often overlooked or undertreated in clinical practice, and effective preventive measures and therapeutic interventions remain limited. Although the exact mechanisms of PSD are not fully understood, emerging evidence suggests that the gut microbiota plays a key role in regulating gut-brain communication. This has sparked great interest in the relationship between the microbiota-gut-brain axis (MGBA) and PSD, especially in the context of cerebral ischemia. In addition to the gut microbiota, another important factor is the gut barrier, which acts as a frontline sensor distinguishing between beneficial and harmful microbes, regulating inflammatory responses and immunomodulation. Based on this, this paper proposes a new approach, the microbiota-immune-barrier axis, which is not only closely related to the pathophysiology of IS but may also play a critical role in the occurrence and progression of PSD. This review aims to systematically analyze how the gut microbiota affects the integrity and function of the barrier after IS through inflammatory responses and immunomodulation, leading to the production or exacerbation of depressive symptoms in the context of cerebral ischemia. In addition, we will explore existing technologies that can assess the MGBA and potential therapeutic strategies for PSD, with the hope of providing new insights for future research and clinical interventions.

## 1 Introduction

Ischemic stroke (IS) is a prevalent central nervous system (CNS) disorder, ranking as the second leading cause of death and the third leading cause of disability worldwide (1). According to the statistics from 2013, there are approximately 25.7 million stroke survivors worldwide, with 71% being patients with IS (2). By 2020, the data showed that IS accounted for about 87% of all stroke cases (3), and the etiology involves a thrombotic or embolic event that leads to impaired blood flow to a region of the brain (4). Post-stroke depression (PSD) is the most common neuropsychiatric comorbidity, affecting more than one-third of IS survivors (5). Patients experiencing PSD often endure cognitive impairment, reduced quality of life, suicidal tendencies, and an increased risk of mortality (6). Despite substantial evidence indicating that PSD is one of the most severe complications following IS, it is frequently overlooked or inadequately treated.

PSD is considered to be a consequence of multiple interactions among biological, psychosocial, and multifactorial factors (7). Cerebrovascular diseases may serve as an initiating or exacerbating factor for depression (8, 9). While the exact pathogenesis of PSD is still not fully understood, its complexity should not be underestimated. According to the gut-brain axis(GBA) theory, alterations in the microbiota are closely linked to changes in brain structure, function, and behavior, and are associated with the pathogenesis of neuropsychiatric disorders (10). In recent years, research has increasingly recognized the gut microbiota as an important modulator of brain development, physiology, and host behavior. The gastrointestinal tract is a major organ for immune response, rich in immune cells, and accounts for over 70% of overall immune system activity (11). The Microbiota alters the intestinal barrier by interacting with immune cells (12), and in certain cases, influences the host by crossing the Blood-Brain Barrier(BBB) through the release of cytokines and metabolites, playing a crucial role in modulating stress-related behaviors, such as depression (13). Therefore, it is essential to explore the impact of the microbiota-gut-brain axis(MGBA) on PSD.

A meta-analysis revealed significant changes in the microbiota composition at the genus, family, and phylum levels in PSD patients compared to healthy individuals (14). Recent studies on animals have demonstrated that adjusting the microbiota can enhance neurological function and alleviate depressive symptoms in PSD rats, simultaneously reinforcing the integrity of the BBB (15). To fully grasp the interactions between the host and its symbiotic partners, it is essential to consider cellular barriers. Increasing evidence indicates that various cellular barriers within the MGBA act as novel conduits linking the microbiota to the brain (16). Traditionally, barriers were viewed as rigid and impenetrable, but it is now acknowledged that cellular barriers are dynamic and meticulously regulated communication interfaces. Consequently, this review will initially explore the interactions among the microbiota, intestinal barrier, and BBB within the MGBA, focusing on immune regulation and inflammatory responses. Next, we will delve into the potential mechanisms by which the microbiota-immune-barrier axis, this MGBA “high-speed pathway,” influences PSD following IS. Finally, to more effectively apply theory to clinical practice, we have thoroughly summarized various detection techniques for the MGBA and potential treatment methods for PSD.

## 2 The intestinal barrier: its structure and function

The gut and brain barriers are fundamentally composed of epithelial or endothelial layers that, under physiological conditions, exhibit varying degrees of permeability. This characteristic is pivotal to their barrier function (17). Nevertheless, it is important to note that this barrier function is not static but rather undergoes dynamic changes. Under physiological circumstances, the primary role of the intestinal barrier is to delineate the body from the external environment, specifically the contents within the gastrointestinal lumen, while simultaneously facilitating the absorption of nutrients. On one hand, the intestinal mucosa acts as a formidable defense, preventing microorganisms from invading the host (18). On the other hand, it also permits symbiotic relationships with certain microorganisms, fostering a harmonious coexistence (19, 20).

The initial line of defense within the gastrointestinal tract is provided by a specialized coating on the exterior of the intestinal epithelium—the mucus layer. This layer is predominantly made up of mucins, notably Muc2, a glycoprotein featuring a network-like structure (21, 22). The mucus layer is divided into an inner and outer layer. It serves as a barrier, preventing large particles and microorganisms from making direct contact with the epithelium, a critical function in minimizing the exposure of intestinal epithelial cells (IEC) to potentially harmful agents (23, 24). Furthermore, the mucus layer is abundant in immunoglobulin A (IgA), secreted by plasma cells (25, 26). IgA facilitates the release of secretory IgA (sIgA) onto the intestinal surface through a complex mechanism known as transcytosis (27, 28). which neutralizes pathogens and aids in sustaining the equilibrium of the symbiotic microbiota (27, 29). It is important to note that the mucus barrier not only serves as a source of nutrients for the microbiota but also offers an ecological niche for their colonization.

The second line of defense is the intestinal epithelial barrier (IEB), which comprises a single layer of columnar epithelial cells (30). This barrier is dependent on cell-cell junctions, where neighboring intestinal cells are linked via junctional complexes, primarily made up of tight junctions (TJs) and adherens junctions, as well as desmosomes (31, 32). TJs are primarily composed of transmembrane proteins, such as claudins and occludins (33), and intracellular proteins, including zonula occludens(ZOs) (34). These structures restrict the diffusion of microorganisms and solutes through the paracellular pathway and dynamically modulate intestinal permeability, which is crucial for preserving the integrity of the epithelial barrier. Additionally, the intestinal epithelium houses several specialized cell types:Goblet cells secrete mucins to sustain the mucus barrier and transport soluble intestinal antigens to dendritic cells (DCs) (35, 36). Microfold cells (M cells), predominantly situated above Peyer’s patches (PPs) in the small intestine (37), facilitate a close antigen-sampling mechanism with DCs (38). Enteroendocrine cells (EECs) secrete various hormones and signaling molecules, acting as a communication bridge between the central and enteric nervous systems (39). Paneth cells produce antimicrobial peptides (AMPs), which regulate both symbiotic and pathogenic bacteria, aiding in the limitation of bacterial resistance and the maintenance of microbial equilibrium (40, 41). Intestinal stem cells (ISCs), located at the base of the crypts, proliferate and differentiate, migrating upwards to replenish various types of IEC (42). IEC can detect microbial stimuli and respond by bolstering their barrier function and coordinating appropriate immune responses, shifting from tolerance to pathogen-specific immunity (43). Consequently, IEC play a pivotal role in the development and homeostasis of mucosal immune cells. In concert with the mucus layer, the IEB controls the ingress of harmful “external” microorganisms into deeper tissues and their dissemination into the circulation.

The third line of defense is the intestinal vascular barrier (IVB), which is comprised of endothelial cells, pericytes, and enteric glial cells (EGCs). These endothelial cells create TJs analogous to those found in epithelial cells, and the IVB serves to shield the body from the passage of harmful molecules through both the IEB and other vascular barriers (44, 45). In contrast to epithelial barriers, intestinal endothelial cells possess a porous structure characterized by small pores delineated by the pore membrane, which enables selective permeability. The creation of these pores is contingent upon a specific endothelial membrane glycoprotein known as plasmalemma vesicle-associated protein-1 (PV-1), which is encoded by the PLVAP gene. PV-1 plays a pivotal role in maintaining endothelial homeostasis and regulating permeability (44, 46).

The gut-associated lymphoid tissues(GALT) is the largest collection of lymphoid tissues in the body, consisting of both organized lymphoid tissues, such as mesenteric lymph nodes and Peyer’s patches (PPs), and more diffusely scattered lymphocytes in the intestinal lamina propria (LP) and epithelium, including large numbers of IgA plasma blasts (47). GALT contains immune cells that coordinate the host’s local and systemic defense against intestinal insults. The LP is a thin layer of loose, non-cellular connective tissue beneath the epithelial layer, rich in immune cells and nerve endings. PPs are dome-shaped structures along the antimesenteric border of the small intestine, featuring lymphoid follicles surrounded by antigen-presenting cells and lymphocytes (predominantly IgA-producing plasma cells). The follicle-associated epithelium of PPs has a thin mucus layer and M cells that facilitate the transport of luminal antigens to the LP (48, 49). T follicular helper cells (Tfh) assist B cells in differentiating into plasma cells, which subsequently produce and secrete sIgA, a classical method (50, 51). These immune cells, including DCs, macrophages(Macs), T cells, and B cells, which are widely distributed in the LP, along with specialized IEC, rapidly respond to the invasion of foreign substances and work together to neutralize inflammation.

**Figure 1:**
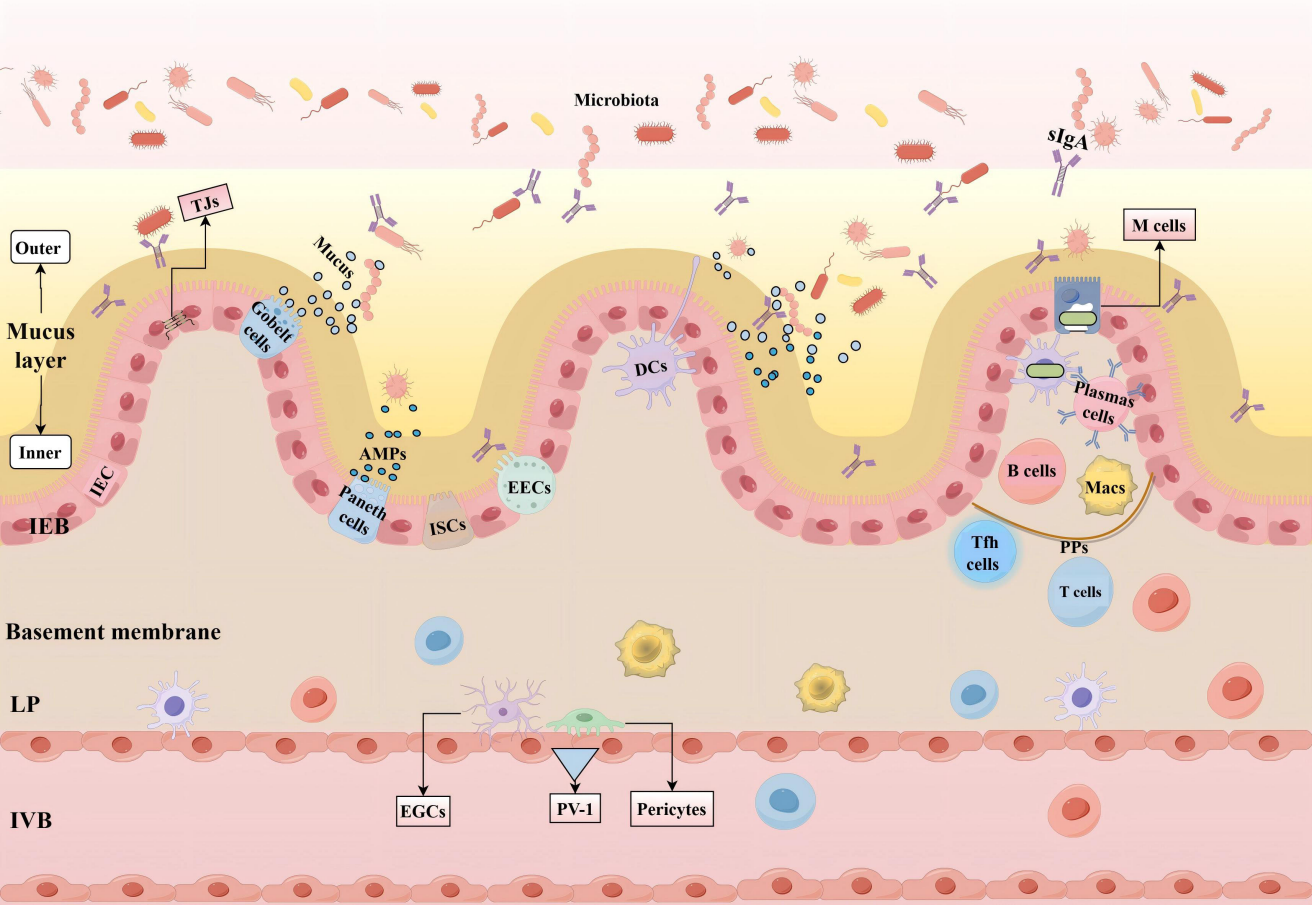
Intestinal barrier and its function. The mucus layer, primarily composed of mucin, is divided into an inner and outer layer and effectively blocks the entry of large particles and microorganisms. The IEB consists of a single layer of columnar IEC, which include various specialized cell types such as goblet cells that secrete mucin, EECs that secrete hormones, Paneth cells that secrete AMPs, and ISCs responsible for regeneration. Adjacent intestinal cells are connected through junctional complexes, including TJs that regulate intestinal permeability. The IVB is composed of endothelial cells, pericytes, and EGCs, which modulate the permeability of the vascular barrier and protect the intestine from harmful molecules. The formation of pores in endothelial cells is regulated by PV-1. GALT includes the LP and PPs, both of which are rich in immune cells, with M cells responsible for transporting antigens and bacteria to DCs. Tfh cells assist B cells in producing and releasing sIgA. IEC interact with DCs, Macs, T cells, and B cells to form a multi-layered defense system that maintains intestinal homeostasis. (IEB, Intestinal Epithelial Barrier; IEC, Intestinal Epithelial Cells; EECs, Enteroendocrine Cells; AMPs, Antimicrobial Peptides; ISCs, Intestinal Stem Cells; TJs, Tight junctions; IVB, Intestinal Vascular Barrier; EGCs, Enteric glial cells; PV-1, Plasmalemma Vesicle-Associated Protein-1; GALT, Gut-Associated Lymphoid Tissue; LP, Lamina Propria; PPs, Peyer’s Patches; M cells, Microfold Cells; DCs, Dendritic Cells; Tfh, T Follicular Helper Cells; sIgA, Secretory Immunoglobulin A; Macs, Macrophages.).

In summary, the mucus layer, IEB, and IVB collectively constitute an intestinal barrier. This barrier possesses chemical, mechanical, and immune properties that interact synergistically to maintain intestinal homeostasis (**Figure 1**).

## 3 Gut microbiota, immune cells, and the integrity and function of the barrier

The gut microbiota consists of various microorganisms, including bacteria, viruses, fungi, and archaea, which coexist symbiotically within the human digestive tract and form a critical part of the gut barrier (52, 53). The microbiota educates the immune system to balance tolerance and defense, thereby maintaining gut homeostasis (54), and affecting distant organs such as the brain (55). In this section, we will delve into the interactions within the Gut Microbiota-Immune Cells-IEC-BBB pathway, as well as the direct and indirect effects of the Gut Microbiota on the barrier.

### 3.1 Gut microbiota-IEC

The importance of interactions between the microbiota and IEC in maintaining the structure and function of the gut barrier has been extensively studied. Specific microbes, such as *Clostridia* and *Bacillus* species, have been shown to effectively induce P-glycoprotein(P-gp) expression in murine IEC, helping to mitigate excessive inflammation and thus maintain gut homeostasis. A positive correlation has also been observed between microbial metabolites, short-chain fatty acids (SCFAs), and P-gp expression (56). Additionally, the microbiota regulates the development and maintenance of EGCs, which are a key component of the IVB (57). EGCs, which are similar to astrocytes in the brain, release soluble factors like S-nitrosoglutathione to regulate the integrity of TJs and support barrier function (58). Studies have shown that capillary network formation is stalled in adult germ-free mice but can resume and develop fully within 10 days of colonization with a complete microbiota or *Bacteroides* (59). Therefore, the microbiota may also influence the IVB directly or through interactions with EGCs. Experiments involving the oral administration of *Lactobacillus casei* and *Lactobacillus paracasei* have shown that these probiotics can increase Paneth cell numbers and enhance the secretion of AMPs, thus bolstering the antimicrobial activity of the intestinal barrier (60). Furthermore, Common SCFAs, including acetate, propionate, and butyrate, serve as energy sources and influence epithelial and immune host cell functions (61, 62). Acetate produced by protective *bifidobacteria* enhances intestinal defense mediated by epithelial cells, thereby protecting the host against lethal infection (63), and *in vitro* studies have shown that *bifidobacterial strains* use acetate to enhance TJs integrity, preventing Tumor Necrosis Factor-alpha(TNF-α)-induced epithelial barrier disruption (64). Acetate also facilitates the production of butyrate by cross-feeding other bacteria (65).

### 3.2 Interactions between IEC and immune cells

IEC expresses a range of pattern recognition receptors (PRRs), which are a diverse and well-characterized class of receptors. These include Toll-like receptors, NOD-like receptors, and RIG-I-like receptors, all of which are integral to innate immunity (66, 67). These receptors have the capacity to recognize pathogen-associated molecular patterns (PAMPs) and damage-associated molecular patterns (DAMPs) (68). While IEC primarily engage in innate immunity, they also play crucial roles in the initiation and modulation of adaptive immune responses. Through the production of cytokines and chemokines, IEC interact with immune cells within the LP and deeper lymphoid tissues. This interaction is indispensable for maintaining immune homeostasis, ensuring a balanced and effective immune response against potential threats.

#### 3.2.1 IEC and IELs

The intestinal epithelial tissue is inhabited by a distinct category of resident immune cells, known as intraepithelial lymphocytes (IELs), which are primarily T lymphocytes. IELs are intimately linked with the intestinal barrier. Initially, the gut microenvironment is abundant in transforming growth factor-beta (TGF-β), which augments the expression of CD103 (αEβ7 integrin) on the surface of IELs. CD103 serves as a pivotal marker for IEL adhesion to E-cadherin, an epithelial cell adhesion molecule, and facilitates the enduring residence of IELs within the epithelial tissue (69, 70). This mechanism facilitates the rapid acquisition of critical signals by IELs from the epithelial tissue and surrounding environment, thereby promoting their homing, maturation, and functional activation. Secondly, interleukin(IL)-15 secreted by IEC fosters the proliferation and survival of IELs and modulates their cytotoxic capabilities. The sustenance of IELs is contingent upon signals emanating from MyD88 and Toll-like receptor 2 within IEC, which are essential for IL-15 production (71). Moreover, chemokine CCL25, produced by IEC, lures CCR9-positive IELs to migrate towards the intestinal epithelium (72). CD8αβ+ IEL located in the epithelial layer of the small intestine have been confirmed to secrete α-defensins, which may serve as an important supplement to the α-defensins produced by Paneth cells. The synergistic effect of CD8αβ+ IEL and Paneth cells can effectively prevent bacterial invasion (73). IELs oversee and preserve the epithelial barrier, engaging in innate immune responses against pathogens. Owing to the absence of genetic tools targeting specific IELs subpopulations, the exact characteristics and mechanisms of IEL functions are not yet fully elucidated.

#### 3.2.2 IEC and ILCs

Innate lymphoid cells (ILCs) are a type of innate immune cells that play a crucial role in regulating the barrier function of various tissues, including the gastrointestinal tract (74). The cytokines secreted by IEC encourage the proliferation and activation of ILCs, encompassing natural killer cells as well as the ILC1, ILC2, and ILC3 subsets. Specifically, ILC1 responds to co-stimulatory signals from IEC, which are mediated by the microbiota, and produces interferon-gamma (IFN-γ) (75), In contrast, ILC2 secretes IL-13 upon infection, targeting crypt ISCs to promote the differentiation of goblet cells (76). Both ILC1 and ILC2 augment goblet cell mucus secretion, thereby aiding in the preservation of intestinal barrier integrity. The activation of ILC2 is contingent upon cytokines derived from epithelial cells, such as IL-25, IL-33, and thymic stromal lymphopoietin (TSLP) (77, 78). ILC3 generates IL-22 (79), which stimulates IEC to produce AMPs, playing a vital role in sustaining epithelial barrier function (80). Additionally, IL-17, produced by ILC3, also contributes to maintaining barrier integrity during intestinal inflammation (81).

#### 3.2.3 IEC and neutrophils

IEC actively contribute to the recruitment of Neutrophils(Neuts) by secreting chemokines. These chemokines, which encompass CXCL7 (82) and CCL20 (83, 84), aid in the migration and infiltration of neuts. Furthermore, matrix metalloproteinase (MMP)-3, produced by IEC, amplifies the bioactivity of CXCL7 (82). During inflammatory episodes, for instance, IL-6, IL-8, and IL-33 derived from IEC play a crucial role in the recruitment and migration of neuts (85–88). Notably, IEC recruit not only neuts but also support their functions by secreting the aforementioned cytokines. Studies have revealed that IL-6 and its soluble receptor sIL-6Rα can regulate the transition of neuts to monocyte infiltration at inflammatory sites by modulating chemokines. In the context of acute inflammation, IL-6 promotes the resolution of neuts, aiding in inflammation resolution; whereas in chronic inflammation, IL-6 increases monocyte infiltration, contributing to disease progression (89). IL-8 not only acts as a direct chemoattractant for neutrophils but also activates neutrophils to release secondary chemokines stored within their granules (90). Furthermore, IL-33 can also induce functional polarization of neuts (91). In summary, IEC play a critical role in regulating neuts recruitment, trans-epithelial migration, cell death, and clearance (92).

As the most abundant type of white blood cells, neuts, like other immune cells, regulate the development and function of IEC. Under physiological conditions, neutrophil-derived IL-22 has been shown to enhance the production of AMPs by IEC, contributing to barrier defense (93). Research has confirmed that neuts also enhance epithelial protection by inducing the production of amphiregulin in IEC through the secretion of TGF-β (94). Under pathological conditions, neutrophil-derived prosecretory factors are closely associated with goblet cell depletion, a histological hallmark of intestinal inflammation (95). It is well-established that the uncontrolled accumulation of overactivated neuts leads to crypt architectural distortion and crypt abscess formation, accompanied by excessive enzymatic reactions, the production of pro-inflammatory cytokines such as TNF-α and IL-1β, and the release of non-cytokine inflammatory mediators such as α-defensins and calprotectin (96, 97). which may be closely linked to the pathogenesis of inflammatory bowel disease.

#### 3.2.4 IEC and DCs/Macs

IEC detect microbial signals and secrete cytokines, including IL-33, TGF-β, and TSLP, which modulate the development of DCs and Macs (98–100). Within the small intestine, IEC generate TGF-β and retinoic acid (RA), facilitating the migration of CD103+ DCs to epithelial cells and expanding the functional repertoire of gut DCs (101). CD103+ DCs reciprocally affect the differentiation of Foxp3+ Tregs by secreting TGF-β and RA (102, 103). Macs can alternate between pro-inflammatory (M1) and anti-inflammatory (M2) states in response to various stimuli (104, 105). M1 macs typically secrete high levels of pro-inflammatory cytokines, such as TNF-α, IL-6, and IL-12. In contrast, M2 macs produce anti-inflammatory cytokines, such as IL-10, which directly or indirectly affect the function of intestinal epithelial cells (106), Additionally, IL-10 helps to promote the expression of Foxp3+ Tregs (107).

#### 3.2.5 IEC and T cells

**Figure 2:**
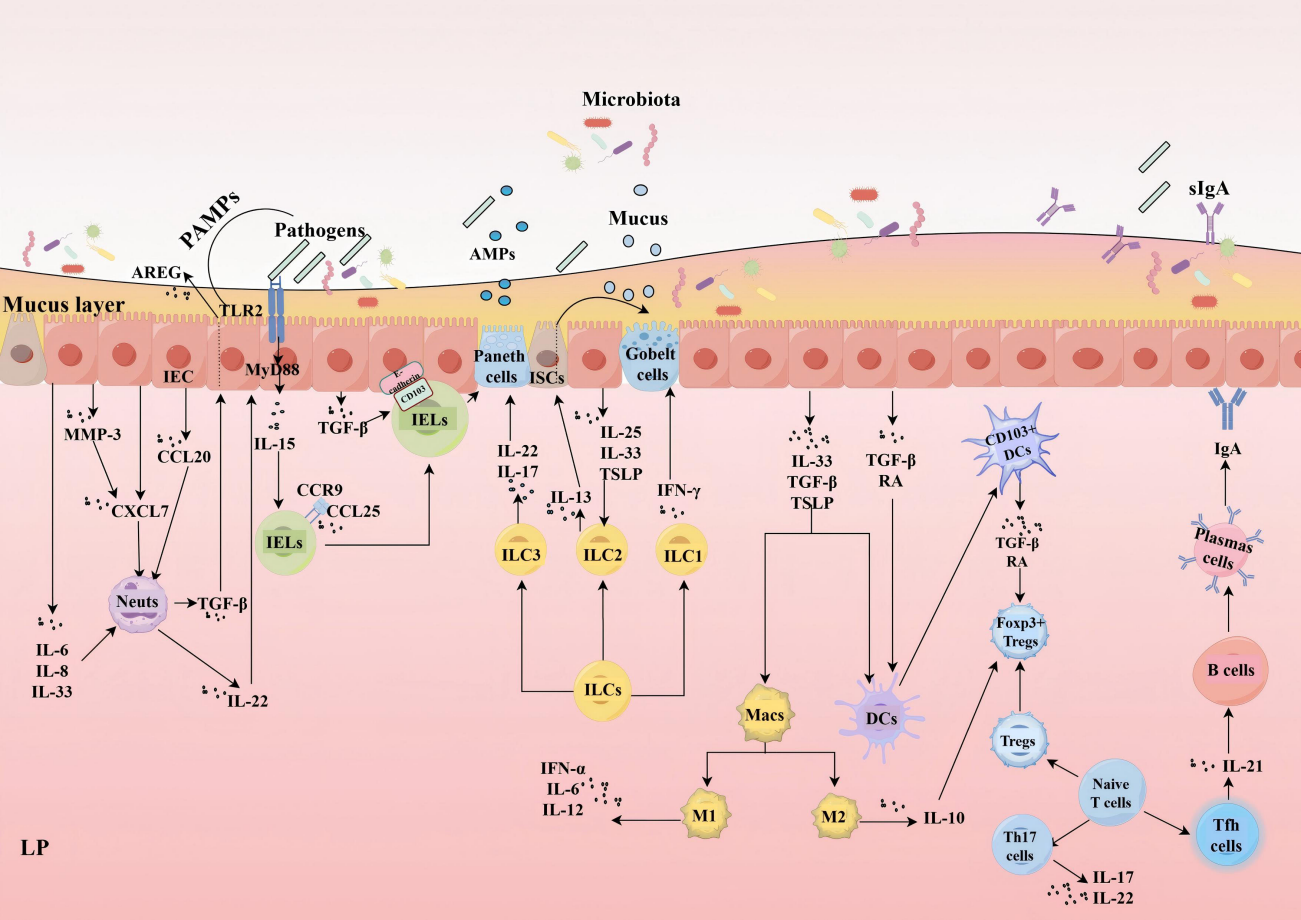
Interactions between IEC and immune cells. IEC promote neuts migration and infiltration by secreting CXCL7, CCL20, and MMP-3, with MMP-3 enhancing CXCL7 activity. During inflammation, IL-6, IL-8, and IL-33 produced by IEC regulate Neuts recruitment and function. Neuts enhance epithelial barrier protection by secreting IL-22 to promote the production of AMPs by IEC and by inducing the generation of AREG in IEC through TGF-β. IELs depend on TGF-β secreted by IEC to induce CD103 binding to E-cadherin, facilitating long-term residency. IL-15, derived from IEC, stimulates the proliferation, survival, and cytotoxicity of IELs; the maintenance of IELs relies on IEC MyD88/TLR2 signaling for the production of IL-15. Additionally, chemokine CCL25, produced by IEC, attracts CCR9-positive IELs to migrate towards the intestinal epithelium. IELs can also work synergistically with Paneth cells to release AMPs.ILC1 responds to signals from IEC to produce IFN-γ; ILC2 secretes IL-13 during infection, promoting crypt stem cell differentiation into goblet cells, both enhancing mucus secretion. ILC2 activation depends on IL-25, IL-33, and TSLP derived from IEC. ILC3-produced IL-22 stimulates IEC to secrete AMPs, while IL-17 maintains barrier function during inflammation. IEC detect microbial signals and secrete IL-33, TGF-β, and TSLP to regulate DCs and Macs. TGF-β and RA promote CD103+ DC migration to epithelial cells; CD103+ DCs reciprocally influence Foxp3+ Tregs differentiation by secreting TGF-β and RA. M1 macs secrete high levels of pro-inflammatory cytokines, such as TNF-α, IL-6, and IL-12. M2 macs produce anti-inflammatory cytokines, such as IL-10, which directly or indirectly affect the function of IEC. Additionally, IL-10 aids in promoting the expression of Foxp3+ Tregs. Th17 cells secrete IL-17 and IL-22, which are vital for maintaining the integrity of the intestinal epithelium. Tfh cells secrete IL-21, which promotes the differentiation of B cells into plasma cells that produce IgA and secrete sIgA. (IEC, Intestinal Epithelial Cells; MMP, Matrix Metalloproteinase; IL, Interleukin; AMPs, Antimicrobial Peptides; Neuts, Neutrophils; AREG, Amphiregulin; TGF-β, Transforming Growth Factor Beta; IELs, Intraepithelial Lymphocytes; ILC, Innate Lymphoid Cell; IFN-γ, Interferon-gamma; TSLP, Thymic Stromal Lymphopoietin; DCs, Dendritic Cells; Macs, Macrophages; RA, Retinoic Acid; Th17,T helper 17 cells; Tregs, Regulatory T cells; TNF-α, Tumor Necrosis Factor-alpha; sIgA, Secretory Immunoglobulin A.).

Effector T cells are typically the dominant lymphocytes in the gut, responsible for mediating a range of host immune defenses and preserving homeostasis. Within the LP, the two most prevalent types are T helper (Th) 17 cells and regulatory T cells (Tregs). These subtypes exhibit heterogeneity. Generally, Th17 cells foster inflammatory immune responses, whereas Tregs suppress excessive or unnecessary immune activation and commonly exhibit anti-inflammatory properties. The functional antagonism between these two subsets is crucial for maintaining immune homeostasis in the LP (108). Th17 cells secrete IL-17, which is pivotal in regulating the integrity of intestinal epithelial and the intestinal mucosal barrier. It influences the cellular distribution of the TJs occludin in IEC (109–111). Furthermore, IL-22 produced by Th17 cells can promote epithelial proliferation and mucosal repair (112). It is important to note that Tfh cells secrete IL-21 which promotes the differentiation of B cells into plasma cells that produce IgA and secrete sIgA (113, 114), thereby fortifying the protection of the epithelial barrier (**Figure 2**).

The gut microbiota-IEC, immune cells-IEC, as well as gut microbiota-immune cells, and the IEC-barrier function, are all key factors affecting barrier function. In short, the stability of the gut barrier depends not only on the self-regulation of IEC but also on the continuous interaction between IEC, immune cells, and the microbiota.

### 3.3 Gut microbiota-immune cells-IEC-BBB

As previously mentioned, the microbial community plays a crucial role through continuous interactions with a range of IEC, shaping the structure of the intestinal barrier and regulating paracellular permeability, which is essential for nutrient absorption and the reinforcement of the mucus layer. This section will explore how the microbiota indirectly modulates immune cells, thereby influencing the structure and function of the barrier, while emphasizing the indispensable role of the coordinated activity of intestinal immune cells.

#### 3.3.1 Gut microbiota-immune cells-IEC

The gut microbiota plays a crucial role in the maturation of the gut mucosal immune system. A review has shown that probiotics not only modulate the expression of mucins and TJs but also influence IEC apoptosis and proliferation, as well as directly or indirectly regulating immune and anti-inflammatory functions. Through these mechanisms, probiotics dynamically maintain the integrity of the intestinal barrier (115). Studies on germ-free mice have shown that they have smaller PPs, immature GALT, reduced intestinal lymphocytes, and lower IgA production (116, 117). However, these damages can be restored by re-establishing the gut microbiota (118). The microbiota’s influence extends beyond the barrier, as microbial metabolites also play critical roles in driving anti-inflammatory and barrier-protective functions, as well as impacting IEC differentiation and gene expression. For instance, butyrate, a metabolite produced by commensal microbes, induces the differentiation of colonic Tregs cells and promotes epithelial integrity (119, 120). The composition of the gut microbiota, especially the composition of *Clostridium* sp*ecies*, may affect the number and function of Tregs and promote the formation of mucosal tolerance (121). *Akkermansia muciniphila* (Am), known as the “intestinal sentinel,” may promote the production of mucin by goblet cells and repair intestinal barrier function (122). Immunologically, Am not only interacts with TLR4 to modify the RoR-T + regulatory T cell immunological response (123), but also activates Macs via the TLR2/NLRP3 signaling pathway both *in vitro* and vivo (124).

In summary, the intestinal barrier is a complex multi-layered structure that includes the microbial barrier. Within this structure, specific microbes act as probiotics; they not only directly affect the barrier but also maintain the balance of mucosal immunity through interactions with immune cells. and disruptions in microbial composition can lead to barrier dysfunction and abnormal substance release along the GBA.

#### 3.3.2 IVB-BBB

The outermost layer of the brain, the meningeal barrier, is located beneath the inner surface of the skull, consisting of the dura mater, arachnoid mater, and pia mater, which encase the brain and cerebrospinal fluid. This layer allows for immune cell transport. Deeper within the brain, there are two critical barriers: BBB and the blood-cerebrospinal fluid barrier, the latter located within the choroid plexus of the brain ventricles (125). The BBB is a highly selective, semipermeable barrier composed of endothelial cells, astrocytic end-feet, and pericytes embedded in the blood vessel basement membrane (126). IVB and the BBB have several similarities, including the increased expression of PV-1 during the injury process and its regulation by the Wnt/β-catenin signaling pathway (127, 128). Furthermore, Wnt/β-catenin signaling in the intestinal endothelium regulates and maintains BBB characteristics during both embryonic and postnatal development: β-catenin enhances endothelial-specific stability to maintain barrier homeostasis, and its inactivation significantly downregulates claudin-3, upregulates vesicle-associated proteins, and leads to BBB disruption (127). A pivotal study revealed that during inflammation, IVB disruption in mice induces choroid plexus vascular barrier closure, restricting access of large molecules (129). Another study suggested that transplantation of EGCs into damaged spinal cords can accelerate the repair of the vasculature and BBB at the injury site (130). These findings suggest that the IVB and BBB are physiologically interconnected and pathologically interrelated.

#### 3.3.3 Gut microbiota-BBB

The human microbiota consists of trillions of microorganisms, including over 1,000 bacterial species and approximately 3 million identified genes, a number 150 times larger than the human genome (131). The primary constituents of the microbiota are the *Firmicutes*, *Bacteroidetes* and *Actinobacteria* (132), When the composition of the microbiota changes, its associated functions may also change or even be compromised (133). The microbiota also plays a vital role in BBB regulation (134). Additionally, these microorganisms have the ability to convert dietary components, such as macromolecules, micronutrients, fibers, and polyphenols, into various metabolites, including SCFAs, trimethylamine, amino acid derivatives, and vitamins. These microbial-derived metabolites play essential metabolic and signaling roles in regulating the host’s internal environment, particularly in terms of their impact on the integrity of the BBB and brain function (13). These studies suggest that the microbiota is essential in regulating the intestinal and brain barriers under physiological conditions. Research indicates that a lack of microbiota is associated with increased BBB permeability and decreased expression of TJs occludin and claudin-5. Transferring fecal matter from pathogen-free mice or treating germ-free mice with SCFAs-producing bacteria can reduce BBB permeability (135). In addition to crossing the BBB and affecting the maturation of microglia, SCFAs also appear to impact neuronal function (136). therefore, SCFAs play a vital role in brain development and CNS homeostasis. An imbalance in the gut microbiota can lead to weakened intestinal barrier function, allowing endotoxins produced by Gram-negative bacteria, such as Lipopolysaccharide(LPS), and harmful substances from opportunistic pathogens to penetrate into the circulatory system. In the case of cerebral ischemia, damage to the BBB enables LPS to enter brain tissue. TLR4 plays a key role in the inflammatory response triggered by LPS, leading to neuroinflammation induced by LPS in microglia/macs, which can further exacerbate ischemic brain injury (137, 138). Furthermore, The gut microbiota plays an important role in Th17 cell differentiation, an important class of CD4+helper T cells, and their infiltration into the brain (139, 140).

In summary, the microbiota impacts BBB integrity through several mechanisms: (1) interactions with a compromised intestinal barrier and immune cells, (2) induction of inflammatory cytokine release by microbial products like LPS, (3) direct regulation of TJs expression by SCFAs or through glial cell modulation neuroinflammation, and (4) stimulation of T cell differentiation and brain infiltration. Therefore, the microbiota and their metabolites have a profound impact on the regulation of barrier function and integrity through their interactions with immune cells. Dysbiosis of the microbiota may lead to barrier dysfunction and abnormal substance release along the GBA, providing a new perspective and understanding for our comprehension of the onset and development of brain diseases.

## 4 IS: gut microbiota, immune cells, and the integrity and function of the barrier

The interaction between IS and the gut microbiota reveals the critical role of the GBA in the pathophysiology of stroke. Based on the close connection between the gut microbiota, immune cells, and integrity and function of barrier function, this section will explore the close relationship between IS and this pathway.

### 4.1 IS-microbiota interactions

Previous studies have confirmed that changes in the gut microbiota can have profound effects on brain. For instance, a systematic review indicated that aging and inflammation might contribute to variations in microbial composition and predispose individuals to IS. The regulation of the Firmicutes/Bacteroidetes ratio could be a potential target for treating IS (141). Another study offers a proof-of-concept demonstrating that the gut microbiome itself is cerebroprotective in experimental stroke (142). However, whether changes in brain function also directly affect the microbiome? The following evidence on the impact of IS on the microbiome may provide an answer. The microbiota of IS patients exhibits significant dysbiosis, characterized by notable alterations in the proportion of *Firmicutes* and *Bacteroidetes*, along with a substantial increase in the abundance of opportunistic pathogens, such as *Enterobacter* and *Desulfovibrio* species (143). Simultaneously, Research has demonstrated the significant impact of brain injury on the composition of the microbiota; these effects include a reduction in the diversity of microbiota species and intestinal bacterial overgrowth, with a preferential expansion of the *Bacteroidetes phylum*. This phenomenon is closely linked to intestinal barrier dysfunction and decreased gut motility, which, when addressed, can lead to improved stroke outcomes (144). Beyond microbiota dysbiosis, there are also prominent changes in its metabolites. Research indicates a decrease in the abundance of SCFAs-producing bacteria, with SCFAs negatively correlated with the severity and prognosis of IS (145, 146). Another metabolite, Tryptophan (Trp), has an index—the ratio of Trp to its competing amino acids in circulation—that is inversely associated with the risk of ischemic stroke (147).

Houlden and colleagues demonstrated that, compared to the sham surgery group, Middle Cerebral Artery Occlusion(MCAO) mice exhibited alterations in the composition of the cecal microbiota, including a significant reduction in *Prevotellaceae* and an increase in *Peptococcaceae*, which correlated with the extent of brain injury and influenced the number of cecal goblet cells and mucin production (148). Beyond preclinical studies, a growing number of clinical investigations are now focusing on changes in the gut microbiota following IS and their association with stroke outcomes. Compared to healthy controls, stroke patients showed a significant increase in *Aerococcaceae(f)*, *ZB2(c)*, *TM7-1(c)*, and *Flavobacterium*, while *Mucispirillum*, *rc4-4*, *Akkermansia*, *Clostridiales(o)*, *Lactobacillus*, and *Stenotrophomonas* were significantly reduced. In terms of functional prognosis afterIS, *Anaerococcus*, *Blautia*, *Dialister*, *Aerococcaceae(f)*, *Propionibacterium*, *Microbacteriaceae(f)*, and *Rothia* were enriched in the group with good prognosis, whereas *Ruminococcaceae(f)* and *Prevotella* were enriched in the group with poor prognosis (149). Yin et al. reported that, compared to asymptomatic individuals, patients with stroke and transient ischemic attack exhibited higher α-diversity (Shannon index) in their microbiota, indicating an increased presence of opportunistic pathogens such as *Enterobacter*, *Megasphaera*, *Oscillibacter*, and *Desulfovibrio*, while symbiotic or beneficial genera like *Bacteroides*, *Prevotella*, and *Faecalibacterium* were relatively less abundant. Furthermore, patients with severe stroke (National Institutes of Health Stroke Scale[NIHSS] score>4) had higher α-diversity indices, more abundant *Proteobacteria*, and fewer *Bacteroides* compared to those with mild stroke (NIHSS score ≤ 4). This microbiota dysbiosis was correlated with disease severity (150). A clinical study on stroke risk stratification revealed that, compared to the low-risk group, the high-risk group exhibited a significantly higher abundance of opportunistic pathogens (e.g., *Enterobacteriaceae* and *Veillonellaceae*) and lactic acid-producing bacteria (e.g., *Bifidobacterium* and *Lactobacillus*), while butyrate-producing bacteria (e.g., *Lachnospiraceae* and *Ruminococcaceae*) were relatively reduced. This suggests that an increase in opportunistic pathogens may be associated with an elevated risk of stroke (151). A new study reveals the intricate interplay between stroke and gut microbiota imbalance. The findings suggest that IS can rapidly lead to gut ischemia and trigger an excessive production of nitrates through free radical reactions, resulting in gut microbiota imbalance. Specifically, the overexpansion and enrichment of *Enterobacteriaceae* exacerbate the condition of cerebral infarction by intensifying systemic inflammatory responses (152). A prospective cohort study revealed no significant differences in α-diversity indices between patients with mild stroke (NIHSS ≤ 3) and non-mild stroke (NIHSS > 4-34). However, significant differences in microbial community composition were observed. Patients with mild stroke exhibited a significant enrichment of *Roseburia*, while those with non-mild stroke showed an enrichment of *Erysipelotrichaceae incertae sedis*. Further analysis demonstrated that the relative abundance of *Roseburia* was significantly correlated with changes in NIHSS scores and short- and long-term functional outcomes, suggesting a potential protective role in stroke development and prognosis. In contrast, the abundance of *Erysipelotrichaceae incertae sedis* was positively associated with stroke severity (153). In patients with acute IS, gut microbiota comparisons between those with favorable outcomes (modified Rankin Scale [RS] score 0-2) and poor outcomes (modified RS score 3-6) at 3 months post-stroke revealed that the poor outcome group was characterized by significantly reduced α-diversity, an increased abundance of pathogenic bacteria (e.g., *Enterococcaceae* and *Enterococcus*), and a decreased abundance of SCFAs-producing bacteria(e.g., *Bacteroidaceae*, *Ruminococcaceae*, and *Faecalibacterium*) (154). Another study found that, compared to healthy individuals, stroke patients exhibited similar gut microbial α-diversity and overall structure. Nevertheless, significant dysbiosis was observed, primarily characterized by an increased abundance of SCFAs-producing bacteria, such as *Odoribacter*, *Akkermansia*, *Ruminococcaceae_UCG_005*, and *Victivallis*. Additionally, *Christensenellaceae_R-7_group* and *norank_f_Ruminococcaceae* were positively correlated with NIHSS1M and RS scores, whereas *Enterobacter* showed negative correlations with both (155) (**Table 1**).

**Table 1:** Changes in gut microbiota following ischemic stroke and their impact on outcomes.

Grouping	Specimen sources	Result	Conclusion	Reference
Sham surgery group vs. MCAO group	Cecal tissue	MCAO group: ↓Prevotellaceae, ↑Peptococcaceae	Changes correlated with the severity of brain injury and affected the number of goblet cells and mucin production in the cecum.	Houlden A, et al., 2016 (148)
Healthy controls vs. IS group	Blood sample	IS group: ↑Aerococcaceae(f), ZB2(c), TM7-1(c), Flavobacterium; ↓Mucispirillum, rc4-4, Akkermansia, Clostridiales(o), Lactobacillus, Stenotrophomonas	Altered microbiota composition may influence IS functional outcomes.	Chang Y, et al., 2021 (149)
Good prognosis group vs. Poor prognosis group	Blood sample	Good prognosis group: ↑Anaerococcus, Blautia, Dialister, Aerococcaceae(f), Propionibacterium, Microbacteriaceae(f), Rothia; Poor prognosis group: ↑Ruminococcaceae(f), Prevotella	Microbiota composition is associated with IS prognosis.	Chang Y, et al., 2021 (149)
Asymptomatic group vs. Stroke and TIA group	Fecal sample	Stroke and TIA group: ↑α-diversity, ↑Enterobacter, Megasphaera, Oscillibacter, Desulfovibrio; ↓Bacteroides, Prevotella, Faecalibacterium	Increased presence of opportunistic pathogens in the patient group.	Yin J, et al., 2015 (150)
Mild stroke (NIHSS ≤ 4) vs. Severe stroke (NIHSS > 4)	Fecal sample	Severe stroke group: ↑α-diversity, ↑Proteobacteria, ↓Bacteroides	Microbiota dysbiosis is correlated with the severity of IS.	Yin J, et al., 2015 (150)
Low-risk group vs. High-risk group	Fecal sample	High-risk group: ↑opportunistic pathogens (e.g., Enterobacteriaceae, Veillonellaceae), ↑lactic acid-producing bacteria (e.g., Bifidobacterium, Lactobacillus); ↓butyrate-producing bacteria (e.g., Lachnospiraceae, Ruminococcaceae)	Increased abundance of opportunistic pathogens may be associated with a higher risk of stroke.	Zeng X, et al., 2019 (151)
Mild stroke (NIHSS ≤ 3) vs. Non-mild stroke (NIHSS > 4–34)	Fecal sample	No significant difference in α-diversity; Mild stroke group: ↑Roseburia; Non-mild stroke group: ↑Erysipelotrichaceae incertae sedis	Roseburia abundance is significantly correlated with NIHSS scores and functional outcomes, suggesting a protective role in stroke development and prognosis. Erysipelotrichaceae incertae sedis abundance is positively associated with stroke severity	Gu M, et al., 2021 (153)
Good outcome group (mRS 0–2) vs. Poor outcome group (mRS 3–6) at 3 months	Fecal sample	Poor outcome group: ↓α-diversity, ↑pathogenic bacteria (e.g., Enterococcaceae, Enterococcus); ↓SCFA-producing bacteria (e.g., Bacteroidaceae, Ruminococcaceae, Faecalibacterium)	Associated with IS outcomes.	Sun H, et al., 2021 (154)
Healthy controls vs. Stroke group	Fecal sample	No significant difference in α-diversity; Stroke group: ↑SCFAs-producing bacteria (e.g., Odoribacter, Akkermansia, Ruminococcaceae_UCG_005, Victivallis)	Christensenellaceae_R-7_group and norank_f_Ruminococcaceae showed positive correlations with NIHSS1M and Rankin Scale scores, whereas Enterobacter showed negative correlations with both.	Li N, et al., 2019 (155)

The ↑ symbol indicates an increase or growth, whereas the ↓ symbol signifies a decrease or reduction.

Although different studies have shown variations in specific microbial changes and α-diversity, the overall trend reveals a strong link between post-stroke gut microbiota dysbiosis and disease severity as well as prognosis. Future research should further conduct large-scale, multicenter studies to validate the complex interactions between gut microbiota and IS, establish causality within specific contexts, elucidate the mechanisms of the GBA, and explore gut microbiota-based intervention strategies, thereby providing new perspectives for the prevention and treatment of IS.

### 4.2 IS-intestinal immune changes

It is well-established that stroke can induce neuroinflammatory responses, a process involving the activation of microglia in the brain (156) and the infiltration of leukocytes (157). The gastrointestinal immune system, a critical immune organ harboring a vast number of immune cells, serves as a significant source of immune cells recruited to ischemic brain tissue (158). Benakis et al. demonstrated that gut microbiota dysbiosis influences the outcomes of IS by suppressing the migration of effector T cells from the gut to the leptomeninges (159). Preclinical studies show that long-term invasion and activation of T cells within the brain have been observed in an experimental model of IS (160). Clinical studies have also found that activated T cells survive in the peripheral blood of IS patients and secrete pro-inflammatory cytokines (161). Further studies have shown that changes in Th1, Th2, and Th17 cells occur within 7 days after an IS. In particular, Th17 cells are associated with the exacerbation of cognitive impairment, recurrence of stroke, and increased mortality in IS patients (162). Recent research has also discovered that stroke triggers extensive lymphocyte apoptosis in intestinal mucosal tissues, particularly B cells and T cells in PPs, leading to a reduction in systemic immunoglobulin levels. Notably, this lymphocyte apoptosis is mediated by neuts extracellular traps released by activated neuts following tissue injury (163). Additionally, lower antibody concentrations in stroke patients may increase susceptibility to bacterial infections (164). Importantly, over 70% of the bacteria detected in infected patients belong to human gut commensals, suggesting that bacterial translocation may occur due to leakage of the intestinal mucosal barrier (165). In summary, impaired intestinal immune function following stroke is both a phenomenon and a critical factor contributing to infections and adverse outcomes.

### 4.3 IS-gastrointestinal barrier complications

In addition to neurological impairments, stroke can also trigger a variety of non-neurological complications, such as gastrointestinal dysfunction, including severe intestinal obstruction, alterations in gut microbiota, and intestinal inflammation. The overactivation of immune cells following stroke is a key factor contributing to intestinal inflammation, which increases intestinal barrier permeability, allowing the translocation of resident microbiota and potential dissemination to systemic organs, thereby predisposing to sepsis (166). Stanley et al. demonstrated that, compared to the sham surgery group, stroke mice exhibited reduced expression of ZO-1, indicating impaired gastrointestinal barrier function and increased intestinal permeability (165). In a mouse model of MCAO, after excluding surgical stress as a potential factor for infection, all mice developed spontaneous bacterial infections within three days. Moreover, over 95% of the cultured bacteria were identified as *Escherichia coli* (167). Another study detected LPS in ischemic brain tissue following stroke (168). A recent meta-analysis by Liu et al. revealed that stroke patients receiving enteral nutrition, including probiotics, had better prognoses and reduced rates of bacterial infections (169). Similarly, another meta-analysis involving 26 randomized controlled trials of probiotic treatment in stroke patients showed that early enteral nutrition combined with probiotics effectively modulated gut microbiota and intestinal mucosal barrier function, enhanced immune responses, and reduced the incidence of infectious complications and gastrointestinal motility disorders (170).

The gut microbiota plays a critical role in the bidirectional communication between the gut and the brain via the GBA, influencing the regulation of key immune cells (171). SCFAs through interaction with free fatty acid receptors, inhibit histone deacetylases and can cross the BBB, affecting microglial function and reducing neuroinflammation, thereby playing a key role in the GBA (172, 173). Studies have shown that SCFAs can reduce neuroinflammation by inhibiting the translocation of LPS to brain tissue, but SCFAs are significantly reduced after IS (174), which adversely affects the regulation of microglia-mediated inflammatory responses. SCFAs not only promote recovery after IS but also protect the intestinal barrier, thereby improving disease prognosis (175). Research has found that fecal transplantation of SCFAs-producing bacteria or SCFAs supplementation can enhance intestinal mucosal integrity and promote the migration of Tregs from the gut to the ischemic brain region (176), reduce neuroinflammation (177), prevent BBB breakdown, and promote neural repair (178). At the same time, SCFAs have also shown effects in improving depression (179). Additionally, Trp metabolites may also influence the occurrence and severity of cerebrovascular diseases. Studies have shown that after IS, levels of Trp and other amino acids are reduced, and a decrease in plasma Trp levels, along with an increase in the kynurenine-to-tryptophan ratio, is associated with depression (180, 181).

In summary, the interaction between IS and gut microbiota profoundly affects stroke pathophysiology and outcomes through the GBA. Dysbiosis of the microbiota, impaired intestinal barrier function, and abnormal immune responses collectively exacerbate stroke-related damage and increase the risk of complications. Notably, the IS-gut microbiota-immune cells-barrier pathway may also influence the development of PSD.

## 5 Examples of techniques used for the microbiota-gut-brain axis

With the deepening of research on the MGBA, a variety of advanced technologies have been widely applied to unravel the complex interactions between the gut and the brain. Below are examples of commonly used techniques and their applications (1): Single-cell RNA sequencing: This technology enables the analysis of gene expression at single-cell resolution, revealing the specific roles of different cell types (182) (e.g., IEC, immune cells, and Glial cells) in the MGBA, Its strength lies in providing high-resolution, cell-type-specific information, which uncovers cellular heterogeneity within the MGBA (183–185). (2) Spatial Transcriptomics: By integrating gene expression data with spatial location information, this technique precisely maps gene expression patterns on tissue sections (186). Its advantage is the ability to reveal spatial distribution of gene expression, aiding in the elucidation of region-specific mechanisms in the MGBA. (3) Multi-omics Integration: This approach combines data from genomics, transcriptomics, proteomics, and metabolomics (187), offering a comprehensive understanding of the intricate interactions within the MGBA. For example, through multi-omics analysis, researchers can explore the interplay among microbial communities, host gene expression, and metabolites, thereby revealing how microbiota dysbiosis impacts brain mood function (188). Its core strength lies in providing a holistic systems biology perspective, facilitating the discovery of multi-level regulatory mechanisms in the MGBA. (4) Optogenetics: This technique utilizes light-sensitive proteins to precisely control the activity of specific neurons, enabling the study of neural circuit functions (189, 190). For instance, by employing these techniques, we can explore the relationship between the gut microbiome and mental illnesses such as schizophrenia (191). Its advantage is the high spatiotemporal resolution in modulating neural activity, shedding light on neural mechanisms within the MGBA. (5) Microbiota Transplantation: By transferring donor microbiota to recipients (e.g., germ-free mice or model mice), this method studies the impact of microbiota on host physiology and pathology, such as the treatment of IS and depression (192, 193). Its strength lies in directly validating the causal role of microbiota, providing a foundation for clinical interventions. (6) *In Vivo* Live Imaging: Utilizing fluorescent labeling and microscopy techniques (194, 195), this technology enables real-time observation of dynamic processes in the gut and brain. Its advantage is the ability to reveal spatiotemporal dynamics within the MGBA. (7) Organoid Models: These models use stem cells to cultivate organoids that mimic the structure of the gut and brain, allowing the study of their functions and interactions (196, 197).The strength of this technology is its ability to provide highly physiologically relevant experimental models, reducing ethical and technical limitations associated with animal experiments. (8) Neuroimaging Combined with Microbiome Analysis: Techniques such as Structural MRI, Functional Neuroimaging, Magnetic Resonance Spectroscopy, and Brain Iron Deposition Imaging, when integrated with microbiome analysis, could investigate the relationship between microbiota and brain microstructure, intrinsic neural activity, functional connectivity, as well as cognitive and emotional functions (198). Its advantage lies in offering non-invasive brain function assessment, combined with microbiome data to elucidate MGBA mechanisms.

The aforementioned technologies represent scientifically robust and practical approaches in current MGBA research. By integrating these techniques, researchers can explore the complex interactions between microbiota and the brain in greater depth, providing strong support for mechanistic studies and therapeutic strategies for diseases such as IS and PSD. The judicious application of these technologies not only advances fundamental research but also offers critical theoretical foundations for clinical translation.

## 6 The link between IS and PSD: microbiota-immune-barrier axis

PSD is the most prevalent neuropsychological disorder among stroke patients, characterized by persistent low mood and diminished interest (199). It is commonly used to describe depressive symptoms following IS, given the predominance of IS in related literature. As early as 2002, Whyte EM and Mulsant BH highlighted in their review that post-stroke depression is not caused by a single biological or psychological factor but rather results from the interplay of multiple factors, aligning with the biopsychosocial model of mental disorders (6). Importantly, a bidirectional relationship exists between depression and stroke: stroke increases the risk of PSD, while depression is an independent risk factor for stroke and stroke-related mortality. In stroke literature, the most consistent finding is that PSD is associated with the severity of stroke and the degree of physical and cognitive impairment (200, 201). Furthermore, studies have shown that the use of antidepressants in PSD patients can improve cognitive function (202), reduce disability (203), and increase survival rates (204).

Presently, discussions on the pathogenic mechanisms and therapeutic targets of major depressive disorder primarily focus on the imbalance of the monoamine neurotransmitter system—which includes serotonin (5-HT), norepinephrine, and dopamine—and the dysfunction of the hypothalamic-pituitary-adrenal (HPA) axis (205). Relatively speaking, PSD is usually triggered by ischemic brain injury, often affecting the frontostriatal and limbic system circuits (206–208), and is accompanied by post-stroke neuroinflammation and impairment of neuroplasticity (209, 210), indicating the presence of structural brain damage. Notably, in studies of IS, Major depressive disorder, or PSD, gut microbial factors are gradually becoming a focal point of research. Studies have found that Major depressive disorder is closely related to changes in the baseline gut microbiota (211), which can regulate Trp metabolism through the GBA and trigger systemic inflammation, serving as significant pathogenic factors (212). PSD may be related to stroke-induced gut microbiota dysbiosis (213), increased gut permeability, and microbial-derived pro-inflammatory metabolites (such as LPS) exacerbating central nervous inflammation (144, 152, 214, 215).

PSD is widely regarded as the result of combined neurobiological dysfunction caused by ischemia and psychosocial distress. However, existing evidence suggests that neurobiological factors (rather than psychological responses to disability) are the primary contributors to PSD (5). In recent years, the gut microbiota has garnered significant attention as a key regulator of the GBA, and its importance in gut-brain communication has expanded the GBA to the MGBA (216). The significance of this axis has become increasingly prominent in research on psychiatry, neurodevelopment, and neurodegenerative diseases. The microbiota and its metabolites communicate with the brain through multiple pathways within the MGBA, thereby influencing brain function and behavior. Based on the analysis in Section 4 of this article, it is evident that IS and the gut microbiota, immune cells, and barrier integrity and functionality exhibit bidirectional interactions. Next, we will further explore how the microbiota-immune-barrier axis affects the occurrence and development of PSD.

### 6.1 IS-MGBA -PSD

The MGBA forms a bidirectional communication network between the microbiota and the host (217). Research has primarily focused on several aspects: neuroanatomical pathways, neuroendocrine pathways of the HPA axis, immune pathways, microbiota metabolic pathways, the intestinal mucosal barrier and BBB (218),. The role of the microbiota in this axis is critical, as various environmental factors and physiological states of the host can influence the composition of the microbiota. When this balance is disrupted, it may lead to microbiota dysbiosis, subsequently affecting the signaling function of the MGBA and adversely impacts the host’s immune, metabolic, and nervous systems (219). It is important to note that these pathways interact and influence each other.

Extensive literature exists on how the microbiota regulates host emotions through MGBA, primarily focusing on the nervous system and neurotransmitters. The brain communicates directly with the gut via parasympathetic and sympathetic fibers and indirectly through the stimulation of the enteric nervous system (220). In this process, enterochromaffin (EC) cells play an significant role. They transmit signals to the brain via the vagus nerve (221, 222). Studies have found that 5-HT synthesized and secreted by EC cells is closely related to the interaction with the microbiota, and in patients with PSD, 5-HT levels are significantly reduced (223). Additionally, γ-aminobutyric acid (GABA), as the major inhibitory neurotransmitter, plays a pivotal role in IS and depression (224). Relevant studies indicate that species such as *Bacteroides*, *Parabacteroides* and *Escherichia* can effectively produce GABA, and the relative abundance of *Bacteroides in feces* is negatively correlated with depression (225). Simultaneously, *Bifidobacterium adolescentis* can produce GABA to modulate the GBA response, and has an intriguing association with depression (226). Furthermore, *Bacillus* members have been demonstrated to boost dopamine production, whereas a rise in *Bifidobacterium* modifies dopamine metabolic abnormalities, improving mood after a stroke (215). The relationship between the microbiota and the HPA axis has also garnered attention. Research reveals that dysbiosis of the gut microbiota can trigger excessive activation of the HPA axis, negatively affecting the development of the prefrontal-limbic circuit. In adult experiments, the use of probiotics can normalize HPA axis activity and alleviate depressive symptoms (227).

**Figure 3:**
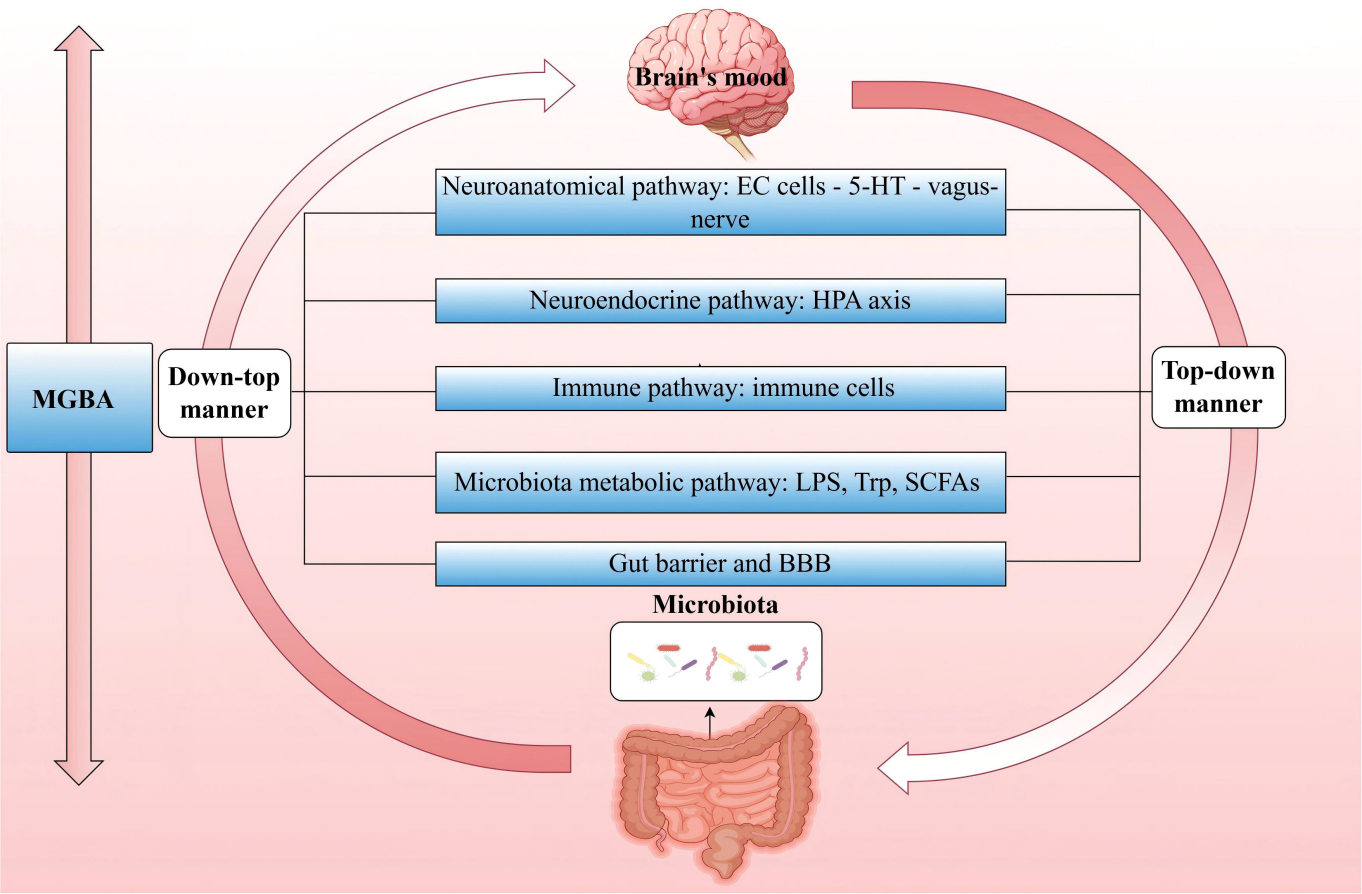
MGBA—Major pathways through which the microbiota regulates host mood. The MGBA represents a bidirectional communication network that interconnects the microbiota with the host. The brain exerts top-down control over the composition and diversity of the microbiota, whereas the microbiota, in turn, exerts bottom-up influence on the brain’s emotional state. (MGBA, Microbiota-Gut-Brain Axis; EC, enterochromaffin; 5-HT, serotonin; HPA, hypothalamic-pituitary-adrenal; LPS, lipopolysaccharide; Trp, Tryptophan; SCFAs, short-chain fatty acids; BBB, blood-brain barrier.).

As mentioned above, the microbiota has a significant impact on central and peripheral immune responses and plays a crucial role in maintaining the integrity of the BBB. Under pathological conditions, dysbiosis of the microbiota can further affect the physiology, behavior, and cognitive functions of the brain through the MGBA, playing a key role in PSD (214, 228). The development and function of the gut immune system largely depend on the microbiota (229), which may potentially play a role in regulating emotions and behavior (222). In the third and fourth parts, we discussed the physiological role of the microbiota, IEC, and immune cells in jointly regulating barrier function, as well as the interaction of this pathway with IS. Next, we will further explore how the microbiota, through immune regulation on the MGBA, affects barrier function post-IS and further influences the host’s emotional state (**Figure 3**).

### 6.2 PSD and the microbiota

A meta-analysis revealed that, compared to healthy individuals, patients with PSD exhibit significant differences in species diversity and microbial community structure at multiple taxonomic levels, including phylum, family, and genus (14). Another study suggested that the gut microbiota may play a role in the pathogenesis of PSD (230). Furthermore, alterations in the composition of the gut microbiota are closely associated with the severity of PSD (231).

Significant changes in the microbiota composition have been observed in PSD patients. Within the *phylum Firmicutes*, there is a reduction in *Bifidobacterium* and an increase in *Enterococcus* and *Escherichia coli* (214), the latter being recognized as an important opportunistic pathogen in the gut (232). Studies have also demonstrated distinct differences in microbiota composition and inflammatory markers between individuals with and without depressive symptoms. Compared to the non-PSD group, the PSD group exhibited higher levels of *Enterococcus faecalis* and *Escherichia coli*, along with elevated inflammatory factors, including IL-1, IL-2, IL-6, and hs-CRP(C-reactive Protein). Concurrently, the PSD group showed lower levels of *Bifidobacterium*. Notably, the levels of *Enterococcus faecalis* and *Escherichia coli* were positively correlated with these inflammatory cytokines, whereas *Bifidobacterium* levels were negatively correlated (214). Another comparative study identified similar microbiota differences between the two groups. Specifically, PSD patients had significantly higher levels of *Streptococcus*, *Akkermansia*, and *Barnesiella*, but lower levels of *Escherichia-Shigella*, *Butyricicoccus*, and *Holdemanella* compared to non-PSD patients. Correlation analysis further indicated that the abundance of *Akkermansia, Barnesiella*, and *Pyramidobacter* was positively associated with Hamilton Depression Scale (HAMD) scores, while the abundance of *Holdemanella* was negatively correlated with HAMD scores (213).

Interventions targeting the microbiota in PSD have been widely reported. As beneficial bacteria, *Bifidobacterium* sp*ecies* inhibit the proliferation of pathogenic bacteria and modulate the microbiota, demonstrating potential antidepressant effects (233). Additionally, *Lactobacillus rhamnosus* has been shown to reduce depression-related behaviors, highlighting the role of the microbiota in emotional regulation (234) (**Table 2**).

**Table 2:** The relationship between PSD and gut microbiota.

Grouping	Result	Conclusion	Reference
Healthy individuals group vs. PSD group	PSD group: Significant differences in species diversity and microbiota structure were observed at multiple taxonomic levels (phylum, family, genus)	The microbiota is closely associated with the occurrence and severity of PSD.	Luo F, et al., 2022 (14); Jiang W, et al., 2021 (230); Ye X, et al., 2021 (231)
Non-PSD group vs. PSD group	PSD group: group: ↑Levels of *Enterococcus faecalis* and *Escherichia coli*, inflammatory factors (including IL-1, IL-2, IL-6, and hs-CRP). ↓*Bifidobacterium*.	Levels of *Enterococcus faecalis* and *Escherichia coli* were positively correlated with these inflammatory cytokines, while *Bifidobacterium* levels showed a negative correlation.	Kang Y, et al., 2021 (214)
Non-PSD group vs. PSD group	PSD group: group: ↑*Streptococcus*, *Akkermansia*, and *Barnesiella*. ↓*Escherichia-Shigella*, *Butyricicoccus*, and *Holdemanella*.	The abundance of *Akkermansia*, *Barnesiella*, and *Pyramidobacter* was positively correlated with Hamilton Depression Scale scores, while *Holdemanella* abundance showed a negative correlation.	Yao S, et al., 2023 (213)
Control group vs. *Lactobacillus rhamnosus* treatment group	Modulation of microbiota composition.	Reduced depression-related behaviors.	Bravo JA, et al., 2011 (234)
Control group vs. *Bifidobacterium* treatment group	Inhibition of pathogenic bacteria proliferation and modulation of microbiota.	Demonstrated potential antidepressant effects.	Tian P, et al., 2020 (233)

The ↑ symbol indicates an increase or growth, whereas the ↓ symbol signifies a decrease or reduction.

### 6.3 PSD and immune regulation

During the early stages of ischemic injury, DAMPs and cytokines expressed at the injury site can enter systemic circulation through the disrupted BBB. This process can trigger immune responses in primary and secondary lymphoid organs, leading to systemic inflammatory response syndrome (235). Among these, the rapid activation of immune cells plays a key role in BBB disruption following IS (236).

Neuroinflammation is known to be associated with CNS disorders, including PSD (237). Inflammatory mediators produced by immune cells play a pivotal role in shaping neuropsychiatric outcomes following stroke. The inflammatory basis of PSD is closely linked to immune cells and molecular factors, with cytokines serving as critical signaling proteins that facilitate intercellular communication. These cytokines are primarily produced by immune cells such as monocytes, macrs, and lymphocytes (238). In the context of PSD, significant elevations in pro-inflammatory cytokines, including IL-1, IL-6, and TNF-α, have been documented (238). Clinical studies have shown that serum levels of TNF-α and IL-1β are elevated in PSD patients compared to non-PSD patients (239). These cytokines can directly affect key brain regions involved in mood regulation, potentially contributing to the development of depressive symptoms. IL-6, synthesized by various cells including neurons, astrocytes, microglia, and endothelial cells, plays a crucial role in the inflammatory response associated with PSD (238, 240). Studies have further emphasized this correlation, demonstrating that higher serum IL-6 levels are independently associated with the occurrence of PSD (241).

Within the immune system, chemokines are responsible for coordinating the migration of cells to specific regions requiring an immune response (238). Particularly, chemokines such as CCL2, CCL7, CCL8, CCL12, and CCL13 have been shown to drive pro-inflammatory cells towards inflamed or injured CNS tissues, playing a significant role in the neuroinflammatory processes associated with PSD (242, 243). Reports indicate that CCL2/CCR2 signaling may be associated with depression (244). Under ischemic conditions, microglia rapidly accumulate at the injury site. They also contribute to tissue repair and remodeling by clearing debris and secreting anti-inflammatory cytokines and growth factors. Conversely, when immune regulation is imbalanced, they exacerbate tissue damage by releasing inflammatory cytokines and neurotoxic substances, highlighting their dual role in the brain’s response to injury (245). Astrocyte activation is a critical response in IS and plays a significant role in the neuroinflammatory environment (246). Following stroke, activated microglia secrete a combination of IL-1α, TNF-α, and C1q, driving astrocytes toward a neurotoxic phenotype, thereby increasing the complexity of the neuroinflammatory response (247). New research indicates that depression is associated with specific networks of the brain’s functional connectome, namely certain brain networks (248, 249). A clinical study revealed that PSD is related to increased functional connectivity strength in specific areas of the default mode network, including the contralateral angular gyrus, posterior cingulate cortex, and hippocampus (250). Further research reveals that most microglia in the PSD hippocampus exhibit both pro-inflammatory and anti-inflammatory states, with a significant negative correlation between IL-1 and PSD (251). It is evident that studying the impact of immune modulation on specific brain circuits in PSD is a field full of potential.

Imbalanced immune regulation may play a key role in the pathophysiology of PSD (252), suggesting that maintaining the homeostasis of immune cells and their mediated cytokines and chemokines in the brain’s inflammatory response is of great significance for the prevention and treatment of PSD.

### 6.4 PSD: barrier integrity and function

Following a stroke, the microvasculature within the affected region exhibits significant inflammatory features, primarily characterized by endothelial dysfunction (253), impaired BBB (254), and the recruitment and infiltration of leukocytes (157). Barrier function impairment can lead to neurological diseases by passive means through the vascular leakage of blood-borne molecules into the CNS, and by active means through guiding inflammatory cells to migrate into the CNS. Both of these mechanisms may be directly related to changes in the molecular composition, function, and dynamics of TJs proteins (255, 256). The invasion of peripheral leukocytes can exacerbate neuronal damage (257, 258). Studies have demonstrated that the protective effects observed in PSD rats are linked to improvements in BBB permeability (259). Moreover, research on depression in mice has revealed that peripheral inflammatory factors can cross the BBB and induce depressive behaviors by modulating BBB integrity, suggesting that the BBB may play a critical role in ameliorating depression in PSD mice (260). Furthermore, in PSD rats, modulation of the gut microbiota has been shown to enhance BBB integrity, improve neurological function, and alleviate depressive symptoms (15). In summary, the destruction of BBB is not only an important pathological process of IS, but also a key factor that may trigger PSD.

### 6.5 IS utilizes the microbiota-immune-barrier axis to influence the occurrence and development of PSD

After a stroke, ecological imbalance, dysregulation of intestinal immune function, and damage to the intestinal barrier become common phenomena (261). Dysbiosis of the gut microbiota not only leads to damage of the intestinal epithelium, reduced mucus secretion, and decreased expression of TJs, thereby increasing intestinal permeability, but also affects neural function and IS outcomes (262–264). Under these conditions, there may be a penetration of ectopic intestinal bacteria and pro-inflammatory cells into brain tissue through a compromised blood-brain barrier (175). It has been confirmed that inflammatory cytokines and other bacterial toxins, such as LPS, penetrate the damaged IEB and enter the circulation (265, 266). Furthermore, studies have found that immune cells, such as Neuts, DCs, Macs, and T cells, infiltrate the brain at different times (267–269). Existing evidence suggests that numerous pro-inflammatory cytokines play a critical role in the development of PSD (270). Under normal physiological conditions, T cells assist B cells in differentiating into plasma cells, which produce IgA to clear toxins and pathogens (271). However, in the MCAO model, early stress leads to significant translocation of gut bacteria and reduced IgA levels (272). Studies have shown that after IS, the host immune system is severely suppressed, and the number of B cells in the small intestine decreases. This may adversely affect the homeostasis of the intestinal and systemic immune systems, impair antimicrobial defenses, and lead to gastrointestinal complications (273). The effects of B cells crossing the damaged BBB and entering brain tissue depend on the subset, timing, and microenvironment (274). It is noteworthy that Th17 cells derived from the small intestine are considered to play a key role in the pathogenesis of depression. They affect the condition by promoting neuroinflammation in the CNS, activating microglia and astrocytes, and inducing neurotoxicity, which is closely related to the onset of depression (275–277). Another important type of T cell, Tregs, secrete anti-inflammatory cytokines to suppress the activity of pro-inflammatory cytokines, promote neurogenesis, and regulate the polarization of microglia and macs after IS (278). Furthermore, the development of depression is a dynamic, multi-stage process involving changes in the response of Tregs to different inflammatory microenvironments (279).

Following IS, the release of DAMPs and cytokines triggers the activation of microglia and astrocytes. Microglia, as the resident immune cells of the CNS, are the first to detect and respond to injury. Within the first 24 hours post-IS, anti-inflammatory M2 microglia dominate (280). During the initial phase of injury, microglia release anti-inflammatory factors to aid in neuronal repair. However, if the injury persists, microglia shift to a pro-inflammatory state, secreting factors that not only exacerbate inflammation but also further damage neurons (238). Ischemic neurons induce the polarization of M1 microglia, which secrete pro-inflammatory mediators, disrupt the BBB, and amplify harmful inflammation (281). In the CNS, astrocytes are the most abundant glial cells and can also be activated into two distinct states post-IS: A1 (pro-inflammatory) and A2 (anti-inflammatory) (282). The cascade of pro-inflammatory mediators and reactive substances released by activated M1 microglia impairs astrocyte function, reduces neurotrophic support, and hinders hippocampal neurogenesis, which is critical for brain repair and cognitive function (283). Studies suggest that the pathological mechanisms of PSD may be linked to reduced miR34b-3p levels in hippocampal neurons and enhanced microglial activation (284). Inflammatory mediators can disrupt extracellular glutamate balance by impairing the glutamate clearance capacity of microglia and astrocytes. This imbalance leads to overactivation of NMDA receptors, excitotoxicity, apoptosis, reduced neuroplasticity, and ultimately neuronal loss, potentially contributing to the development of PSD (285, 286).

**Figure 4:**
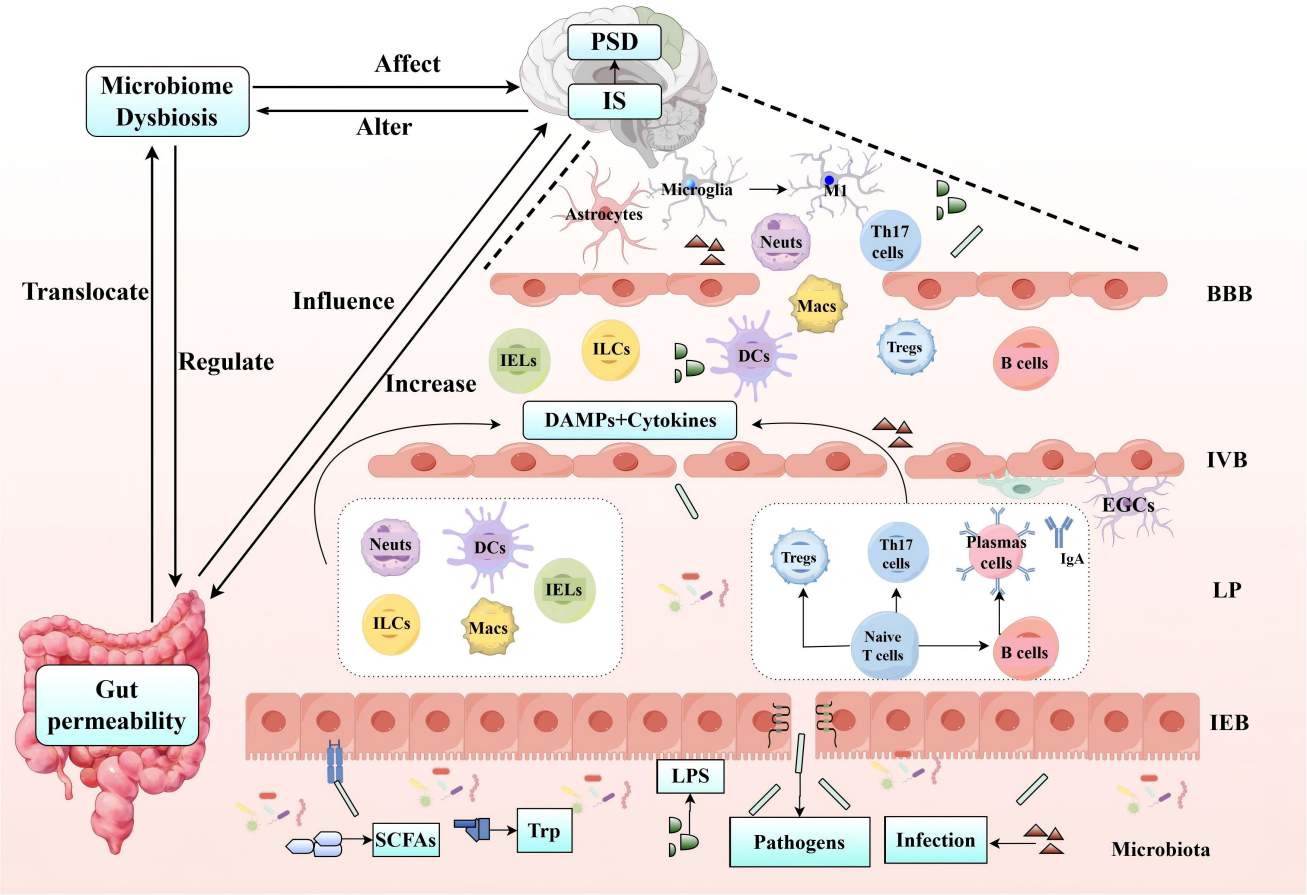
IS utilizes the microbiota-immune-barrier axis to influence the occurrence and development of PSD. Following IS, microbiota dysbiosis exacerbates intestinal epithelial barrier (IEB, IVB) dysfunction, resulting in the translocation of harmful substances across the intestinal barrier and the excessive release of local inflammatory cytokines. This process triggers immune regulation in the LP, involving both innate immune cells (such as Neuts, IELs, ILCs, DCs, and Macs) and adaptive immune cells (including Th17 and Tregs within the T cell population). The subsequent release of DAMPs and cytokines further amplifies the inflammatory response, leading to the activation of microglia and astrocytes, the secretion of pro-inflammatory mediators, disruption of the BBB, and the development of PSD. (IS, Ischemic Stroke; IEB, Intestinal Epithelial Barrier; IVB, Intestinal Vascular Barrier; Neuts, Neutrophils; IELs, Intraepithelial Lymphocytes; ILCs, Innate Lymphoid Cell; DCs, Dendritic Cells; Macs, Macrophages; Th17, T helper 17 cells; Tregs, Regulatory T cells; PSD, Post-Stroke Depression).

In summary, following IS, microbiota dysbiosis exacerbates intestinal barrier dysfunction, leading to the excessive release of local inflammatory cytokines (287). This activates immune regulation and intensifies the inflammatory response, affecting the homeostasis of both the intestinal and brain barriers. In the context of ischemia, the disruption of the microbiota-immune-barrier axis in the MGBA further promotes the development and progression of PSD (**Figure 4**).

## 7 Potential therapeutic strategies for PSD—modulating the gut microbiota

The intricate nature of PSD pathophysiology renders biological prevention and treatment approaches particularly challenging. Presently, treatment strategies for PSD predominantly encompass pharmacological therapy, neurostimulation, and psychological interventions. Although selective 5-HT reuptake inhibitors have demonstrated clinical significance, their efficacy is still debated, and they come with risks, such as the potential for bleeding (5). The prolonged use of antidepressants, the risk of dependency, and a range of side effects have steered interest towards alternative treatments. Therapies based on microbiota, which have the potential to simultaneously address the underlying condition and alleviate depressive symptoms, may emerge as a central focus in future research endeavors.

### 7.1 Probiotics and prebiotics

Probiotics are a class of safe microorganisms that can bring numerous benefits to the host when given to human subjects in adequate doses and at the right time (288). Reportedly, Probiotics enhance barrier function by increasing mucus production, AMPs, and sIgA levels, promoting competitive adherence against pathogens, and improving the TJs integrity of IEC (289). Preclinical studies reveal that *Lactobacillus rhamnosus* and *Bifidobacterium breve* show potential in improving neurological dysfunction caused by MCAO in rats by inhibiting neuroinflammation and modulating the GBA (290). Clinical research indicates that consuming probiotics can help enhance patients’ emotional well-being, particularly alleviating symptoms of depression and anxiety that manifest within three months following a stroke (291). Another clinical study has found that tablets containing a combination of live *Bifidobacterium*, *Lactobacillus*, *Enterococcus*, and *Bacillus cereus* can promote neurological recovery and alleviate depression in stroke patients. These effects may be attributed to the regulation of NF-κB, IL-1β, and TNF-α levels (292). Another meta-analysis showed that the combined use of probiotics with enteral nutrition significantly reduced the levels of TNF-α, IL-6, and IL-10, and statistically significantly decreased the incidence of pulmonary, gastrointestinal, and urinary tract infections, mortality, and the occurrence of intestinal dysbiosis (293).

Prebiotics show great potential in altering the gut microbiota, with different prebiotics promoting the growth of different native gut bacteria (294). Research has found that lactulose can improve neurological function after stroke by inhibiting harmful bacteria, correcting metabolic disorders, repairing damaged intestinal barriers, and suppressing inflammatory responses in mice after stroke (295). Furthermore, a fiber-rich barley variety known as BARLEYmax has been shown to increase butyrate levels in the gastrointestinal tract, thereby promoting the proliferation of beneficial bacteria (296). Similarly, dietary fiber inulin has been observed to reshape the microbiota in mice, enhancing intestinal barrier integrity through the upregulation of TJs protein expression and increasing SCFAs in feces. This nutritional intervention strategy may prevent depression symptoms by leveraging the microbiota-gut-SCFAs axis (297). In summary, the supplementation of probiotics or prebiotics can regulate the microbiota, thereby affecting the stability of the intestinal barrier and ultimately influencing brain function, offering a novel approach for the treatment of PSD.

### 7.2 Fecal microbiota transplantation

Fecal microbiota transplantation (FMT) involves the transfer of fecal matter from a healthy donor into the gastrointestinal tract of a patient to treat specific diseases (298). The advantages of FMT have been acknowledged since the fourth century, during the Eastern Jin Dynasty in China. Research has demonstrated that FMT can prevent ischemic injury by reducing the expression of IL-17, IFN-γ, and other pro-inflammatory cytokines (299). Transplanting fecal bacteria rich in SCFAs and supplementing with butyric acid have been found to be effective treatments for IS (300). Another study indicates that FMT improves depressive-like behavior, corrects gut microbiota imbalance, and alleviates intestinal tract inflammation, intestinal mucosa disruption, and neuroinflammation in rats induced by chronic unpredictable mild stress (301). Consequently, FMT may represent a potential therapeutic approach for PSD. Further investigation into the mechanisms underlying FMT, including refining donor screening processes, optimizing fecal preparation techniques, and exploring alternative administration routes, may enhance its efficacy and safety.

### 7.3 Vagus nerve stimulation

Vagus nerve stimulation (VNS) is an approved method for treating epilepsy and is currently being researched for application in the treatment of other diseases, including depression, anxiety disorders, and Alzheimer’s disease (302). Previous research has reported that VNS can prevent intestinal permeability induced by traumatic brain injury. Additionally, VNS enhances enteric glial activity, potentially mediating the CNS’s regulation of intestinal permeability (303). Recent animal research has revealed that VNS ameliorates microbiota imbalance and mitigates BBB damage in rats with MCAO via the MGBA (304). Clinical studies reveal that VNS therapy can alleviate the damage to the BBB and colonic barrier after cerebral ischemia/reperfusion by modulating immune cells, and mitigate systemic inflammatory responses (305). In recent years, non-invasive transcutaneous auricular VNS (ta-VNS) has garnered interest, indicating that ta-VNS triggers anti-inflammatory pathways, restores MGBA homeostasis, and modulates psychiatric disorders (306). Additional studies have observed that ta-VNS increases the abundance of *lactobacilli* and *bifidobacteria* (307). Double-blind, randomized controlled trials have shown that the synergistic approach of combining ta-VNS with conventional treatment demonstrates remarkable efficacy and tolerability in managing PSD (308). In summary, ta-VNS represents a safe and efficacious novel therapeutic approach.

### 7.4 Traditional Chinese medicine (herbal medicine, acupuncture)

The traditional Chinese herbal extract Gastrodin (Gas), derived from the herb Tianma, has been studied extensively. Research indicates that Gas enhances intestinal barrier function by increasing the expression of TJs proteins and mucins. Additionally, it significantly reduces the secretion of pro-inflammatory cytokines in mice (309). Further studies have demonstrated its efficacy in alleviating behavioral deficits associated with depression and suggest its potential in the prevention and treatment of PSD (310). Moreover, Gas influences the gut microbiota and has been shown to improve depressive-like behaviors in mice (311). A bibliometric analysis reveals that from 2014 to 2023, numerous researchers have persistently investigated the role of acupuncture in PSD (312), corroborating its positive impact on depression (313). Acupuncture can address PSD through various mechanisms, including the protection of the intestinal mucosal barrier, immune regulation, and inflammation control, with the modulation of the gut microbiota being a common underlying theme (314). The study also found that acupuncture can alleviate depressive-like behavior in PSD by regulating the gut microbiota and inhibiting the overactivation of inflammatory mediators (315). Acupuncture can also effectively promote the rehabilitation process of PSD patients by maintaining the dynamic balance of gut microbiota, thus proving that acupuncture, as a non-pharmaceutical treatment, has significant potential in alleviating depressive symptoms (314). Moreover, acupuncture, whether administered as a standalone therapy or in conjunction with other treatments such as music therapy and repetitive transcranial magnetic stimulation, has been evidenced to effectively alleviate depressive symptoms (316–318). Despite the promising outlook for acupuncture in the treatment of PSD, further exploration is warranted to fully understand its potential mechanisms and clinical efficacy.

In the treatment of PSD, non-pharmacological interventions such as acupuncture and ta-VNS have garnered significant attention. However, their widespread application faces challenges, including unclear mechanisms and insufficient clinical evidence. Moving forward, through multidisciplinary collaboration and technological innovation, these therapies hold promise as integral components of PSD treatment, offering safer and more effective options for patients.

## 8 Conclusion

IS remains one of the significant global health challenges, with one of its complications-PSD-urgently requiring more attention. Currently, the exact pathogenesis of PSD, particularly its interaction mechanism with gut microbiota, is not fully understood. This review delves into how post-stroke gut microbiota dysbiosis leads to barrier dysfunction through complex immune regulation and inflammatory responses, and proposes the concept of a microbiota-immune-barrier axis based on the MGBA. The article elaborates on the connection between this pathway and IS as well as PSD, aiming to provide new insights and perspectives on the potential pathogenesis of PSD to promote the development of clinical prevention and treatment strategies for PSD. Although We have organized and summarized the potential pathogenesis of PSD, considering the heterogeneity of microbiota, immune cells, and patients, as well as the dynamic changes in the stages of PSD, it is currently impossible to establish a single definitive causal relationship. This is precisely the challenge that future research needs to further address.
